# The Neuronal Actions of Leptin and the Implications for Treating Alzheimer’s Disease

**DOI:** 10.3390/ph14010052

**Published:** 2021-01-11

**Authors:** Kirsty Hamilton, Jenni Harvey

**Affiliations:** Systems Medicine, Ninewells Hospital and Medical School, University of Dundee, Dundee DD1 9SY, UK; k.u.hamilton@dundee.ac.uk

**Keywords:** Leptin, hippocampus, synaptic plasticity, tau, AMPA, Alzheimer’s, amyloid, memory, synaptic transmission

## Abstract

It is widely accepted that the endocrine hormone leptin controls food intake and energy homeostasis via activation of leptin receptors expressed on hypothalamic arcuate neurons. The hippocampal formation also displays raised levels of leptin receptor expression and accumulating evidence indicates that leptin has a significant impact on hippocampal synaptic function. Thus, cellular and behavioural studies support a cognitive enhancing role for leptin as excitatory synaptic transmission, synaptic plasticity and glutamate receptor trafficking at hippocampal Schaffer collateral (SC)-CA1 synapses are regulated by leptin, and treatment with leptin enhances performance in hippocampus-dependent memory tasks. Recent studies indicate that hippocampal temporoammonic (TA)-CA1 synapses are also a key target for leptin. The ability of leptin to regulate TA-CA1 synapses has important functional consequences as TA-CA1 synapses are implicated in spatial and episodic memory processes. Moreover, degeneration is initiated in the TA pathway at very early stages of Alzheimer’s disease, and recent clinical evidence has revealed links between plasma leptin levels and the incidence of Alzheimer’s disease (AD). Additionally, accumulating evidence indicates that leptin has neuroprotective actions in various AD models, whereas dysfunctions in the leptin system accelerate AD pathogenesis. Here, we review the data implicating the leptin system as a potential novel target for AD, and the evidence that boosting the hippocampal actions of leptin may be beneficial.

## 1. Introduction

It is over 50 years since Hervey performed ground-breaking parabiosis experiments which suggested that a circulatory factor regulates food intake and body weight [1]. This factor has since been identified as the 16 kDa *ob* gene product, leptin, which is derived from the Greek word leptos, meaning thin [2]. The major site for the production of leptin is white adipose tissue, although leptin is also made in various other peripheral tissues, including skeletal muscle, placenta and mammary epithelium. The circulating plasma levels of leptin directly correlate to overall body fat content [3]. Although leptin is predominantly produced by peripheral tissues, it readily gains entry to the brain via a saturable transport process at the blood–brain barrier or via the cerebrospinal fluid [4]. Within the CNS, specific hypothalamic nuclei such as the arcuate nucleus and ventromedial hypothalamus are key targets for leptin. After a meal, circulating leptin levels increase, leading to a rise in the CNS levels of leptin which in turn signals a feeling of fullness. In contrast, after periods of starvation or fasting, the circulating leptin levels fall, which acts as a stimulus for feeding [5]. 

Studies in this area have been aided by the availability of rodents with naturally occurring mutations in the leptin and lepR genes. Mutations in the *ob* gene have been shown to underlie early-onset obesity, hypothermia, and hyperphagia in mice. Thus, in *ob*/*ob* mice, there is a lack of leptin as a truncated leptin protein is generated that is unable to be secreted. In a different leptin-deficient mouse strain (*ob*^2J^/*ob*^2J^), a transposon is integrated into the *ob* gene, thereby preventing *ob* mRNA synthesis. Interestingly, *ob* gene mutations rarely occur in humans. However, cases of morbid obesity identified in families from Pakistan and Turkey have been linked to specific mutations in the *ob* gene. The low incidence of leptin-specific mutations in humans indicates that genetic abnormalities in the leptin system are not the principal route for the development of obesity in humans. 

## 2. Leptin Receptors

### 2.1. Leptin Receptor Isoforms

Leptin produces its biological effects via activating the leptin receptor (LepR). Tartaglia and colleagues first isolated LepR from the choroid plexus, and six isoforms of the receptor have been identified (LepRa-f) [6]. The isoforms differ with respect to their C-terminal domain length, which in turn influences the signalling capacity of the different LepR isoforms. It is widely accepted that the long-form (LepRb) is the predominant signalling competent isoform due to the extended length of its C-terminal domain. Conversely, the short isoforms (LepRa,c,d,f) have shorter C-terminal domains and limited signalling capacity. LepRe is distinct as it lacks a transmembrane domain but it is thought to be a route for transporting leptin in the plasma, as it still retains leptin-binding capacity. 

### 2.2. Leptin Receptor-Driven Signalling

**Figure 1:**
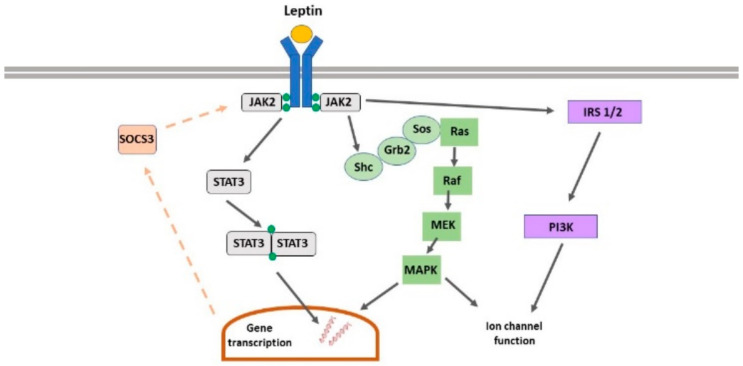
Leptin receptor-driven signalling in neurons. The key signalling pathways activated following leptin binding to leptin receptors (LepRs) and subsequent activation of janus tyrosine kinase (JAK)2 are shown including signal transducers and activators of transcription (STAT3), Ras-Raf-MEK-MAPK and phosphoinositide 3-kinase (PI3K), which ultimately lead to gene transcriptional changes or altered ion channel function. LepR signalling is terminated following the production of suppressor of cytokine signalling (SOCS)3.

LepRs are members of the class I cytokine receptor superfamily which signal via association with janus tyrosine kinases (JAKs) [7]. Leptin binding to its receptor promotes phosphorylation and subsequent activation of JAK2, leading to recruitment and stimulation of various downstream pathways including phosphoinositide 3-kinase (PI3K), ERK, and signal transducers and activators of transcription (STAT3). LepR signalling can be terminated via activation of SOCS1-3 (suppressor of cytokine signalling), which bind to phosphorylated JAK2, thereby limiting LepR-driven signalling [8]. In hippocampal neurons, activation of PI3K, ERK and STAT3 are implicated in LepR-driven regulation of synaptic function (Figure 1) [9]. 

In rodents, LepR gene mutations result in leptin-insensitivity, which ultimately leads to development of early-onset obesity as well as several endocrine-related impairments [2]. In *db*/*db* mice, a truncated form of LepR is produced which is unable to signal via the conventional JAK-STAT signalling pathway. Conversely, in *fa*/*fa* rats, a single point mutation occurs that renders all the LepR isoforms signalling incompetent [2]. In a manner similar to the *ob* gene mutations, the incidence of human LepR gene mutations is also extremely low.

### 2.3. Leptin Receptor Expression in the CNS

In accordance with leptin’s pivotal role in controlling energy homeostasis, LepRs are highly expressed in specific regions of the hypothalamus that govern food intake and body weight, such as arcuate nucleus and ventromedial hypothalamus [10,11]. Additionally, high levels of LepR expression are observed in many other brain regions, including the hippocampus and cortex [12,13]. This pattern of LepR expression in the CNS suggests potential important roles for leptin in higher brain functions. Indeed, early studies involving leptin-deficient (*ob*/*ob*) mice demonstrated that absence of leptin resulted in neurodevelopmental abnormalities, with specific impairments detected in hippocampal and cortical brain regions [14]. These deficits were readily reversed by leptin replacement, suggesting a role for leptin in neuronal development. In support of this hypothesis, a surge in leptin is observed during the critical period of postnatal development [15]. Subsequent studies in this area have identified that this postnatal leptin surge plays a critical role in development of key hypothalamic synaptic connections involved in control of energy homeostasis [16]. However, increasing evidence indicates that the modulatory effects of leptin are not restricted to early postnatal stages of development, as leptin is reported to markedly influence the functioning of the hippocampus in adulthood and during ageing. Thus, in adult, tissue treatment with leptin has a major impact on the cellular processes underlying hippocampal learning and memory, which has led to the proposal that this hormone acts as a potential cognitive enhancer [17,18]. In support of this possibility, deficits in hippocampus-specific processes, such as spatial learning and memory, accompany insensitivity to leptin [19,20]. 

## 3. Leptin Regulation of Hippocampal Excitatory Synaptic Function

### 3.1. SC-CA1 Synapses

Immunocytochemical studies performed on cultured hippocampal neurons provided the first experimental evidence for expression of LepRs at hippocampal synapses [21], as LepR-positive immunolabelling was not only detected on principal CA1 neurons but also co-localised with the synaptic marker, synapsin-1. Subsequent electrophysiological studies observed that application of leptin depressed excitatory synaptic transmission at Schaffer-collateral (SC)-CA1 synapses in acute hippocampal slices obtained from juvenile (P11-18) rats [22]; an effect that was readily reversed on washout of leptin. The ability of leptin to reversibly depress hippocampal excitatory synaptic transmission at SC-CA1 synapses has been observed in mouse and rats at similar developmental stages [23,24]. In P5-8 hippocampal slices, leptin induces a novel form of *N*-Methyl-d-aspartate (NMDA) receptor-dependent long-term depression (LTD) [25]. In contrast, in adult hippocampus (12–16 weeks), leptin has an opposing effect as it results in a long-lasting increase in excitatory synaptic transmission that persists even after washout of leptin [25]. The ability of leptin to induce long-term potentiation (LTP) at adult SC-CA1 synapses also requires NMDA receptor activation as the effects of leptin are blocked by the competitive NMDA receptor antagonist D-AP5 [24,25]. 

It has been hypothesised that activation of NMDA receptors comprised of different subunits underlie different forms of activity-dependent synaptic plasticity. Indeed, the use of specific pharmacological tools to block different NMDA receptor subunits revealed that LTP requires stimulation of GluN2A subunits, whereas GluN2B subunits are implicated in LTD [26,27]. Similarly, inhibition of GluN2B subunits with either ifenprodil or Ro25-6081 blocked leptin-induced LTD at P5-8 hippocampal SC-CA1 synapses, suggesting a crucial role for GluN2B subunits in this process. Activation of GluN2A subunits is also implicated in leptin-induced LTP at adult SC-CA1 synapses as treatment of slices with the putative GluN2A antagonist, NVP-AAM077 completely blocked the ability of leptin to induce LTP [24]. In addition to the involvement of distinct NMDA receptor subunits, divergent signalling cascades are implicated in the bi-directional effects of leptin on synaptic efficacy. Thus, activation of ERK signalling is pivotal for leptin-induced LTD, whereas leptin-induced LTP in adult hippocampus requires stimulation of the PI3-kinase pathway [24]. 

### 3.2. TA-CA1 Synapses

In addition to the SC input, hippocampal CA1 neurons are directly innervated by the temporoammonic (TA) pathway, which extends from layer III of the entorhinal cortex and forms synapses within the stratum-moleculare region of CA1. TA-CA1 synapses are known to express high levels of dopamine receptors, and this feature can be used in electrophysiological studies to readily identify TA-CA1 synaptic connections. Thus, stimulation of TA-CA1 synapses can be verified by applying dopamine, which markedly depresses excitatory synaptic transmission at TA-CA1 synapses but has no effect at SC-CA1 synapses [28,29]. Recent evidence indicates that in a manner similar to SC-CA1 synapses, TA-CA1 synapses are also rapidly modulated by the hormone leptin. Thus, treatment of juvenile hippocampal slices with leptin results in the induction of a novel form of LTP at TA-CA1 synapses [29]. This contrasts with the synaptic depression induced by leptin at SC-CA1 synapses at the same stage of development. However, both these forms of leptin-driven synaptic plasticity require NMDA receptors and specifically activation of GluN2B subunits [29] synapses as leptin-induced LTP at juvenile TA-CA1 synapses is mediated by PI 3-kinase, not ERK, signalling. 

In contrast to SC-CA1 synapses, at adult TA-CA1 synapses leptin has the ability to induce a novel form of LTD, a process that is NMDA receptor-dependent and specifically requires activation of GluN2A-containing NMDA receptors [30]. Moreover, activation of canonical JAK2-STAT3 signalling and subsequent endocytosis of α-amino-3-hydroxy-5-methyl-4-isoxazolepropionic acid (AMPA) receptors comprising GluA1 subunits is necessary for leptin-induced LTD. This novel form of LTD shows parallels to activity-dependent LTD induced at SC-CA1 synapses, as JAK-STAT signalling is also implicated in SC-CA1 LTD [31]. However, there are clear differences in the role of JAK-STAT3 in these two forms of LTD as gene transcriptional changes underlie leptin-induced LTD at TA-CA1 synapses, whereas NMDAR-dependent LTD induced at SC-CA1 synapses is independent of gene transcriptional changes. 

### 3.3. Leptin Regulation of AMPA Receptor Trafficking

It is widely recognised that synaptic insertion and removal of specific AMPA receptor subunits play a key part in activity-dependent synaptic plasticity in the hippocampus [32]. Transient synaptic incorporation of GluA2-lacking AMPA receptors has also been reported to accompany hippocampal LTP in some studies [33,34]. In accordance with the involvement of AMPA receptor trafficking in activity-dependent LTP, the leptin-driven alteration in synaptic efficacy at adult SC-CA1 synapses involves synaptic insertion of GluA2-lacking AMPA receptors [25]. Thus, leptin-induced LTP is reversed by addition of specific inhibitors of GluA2-lacking AMPA receptors, namely, philanthotoxin. In whole-cell recordings, leptin-induced LTP is also associated with an increase in the rectification index of synaptic AMPA receptors, suggesting incorporation of GluA2-lacking AMPA receptors mediates leptin-induced LTP. In parallel studies performed in cultured hippocampal neurons and in acute brain slices, exposure to leptin culminates in an increase in the plasma membrane expression as well as the synaptic expression of the AMPA receptor subunit, GluA1 [25]. Together, these findings suggest that the ability of leptin to induce LTP at adult SC-CA1 synapses involves trafficking of GluA2-lacking AMPA receptors into hippocampal synapses. 

**Figure 2:**
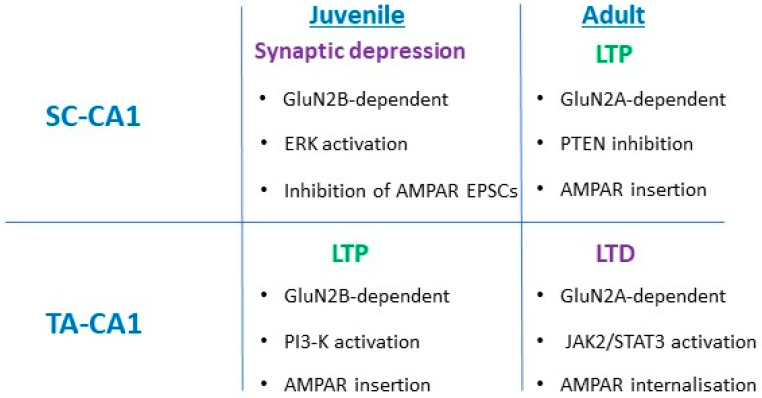
Summary of the differential effects of leptin at Schaffer collateral (SC)-CA1 and temporoammonic (TA)-CA1 synapses. Treatment of juvenile hippocampus with leptin results in a transient synaptic depression at SC-CA1 synapses that involves GluN2B activation and stimulation of ERK. Conversely, leptin induces LTP at juvenile TA-CA1 synapses, which requires GluN2A activation and PI 3-kinase-driven synaptic insertion of GluA2-lacking AMPA receptors. At adult SC-CA1 synapses, leptin induces a novel form of LTP that is also NMDA receptor-dependent and involves synaptic insertion of GluA2-lacking AMPA receptors following inhibition of PTEN. In contrast, leptin induces long-term depression (LTD) at adult TA-CA1 synapses, and this process requires GluN2A activation and JAK2/STAT3 signalling, which results in AMPA receptor removal from synapses.

In a similar manner, trafficking and insertion of AMPA receptors into synapses play a pivotal part in leptin-induced LTP at juvenile TA-CA1 synapses [26], as prior treatment with philanthotoxin blocked the ability of leptin to induce LTP. The leptin-driven persistent increase in synaptic transmission was also reversed by addition of philanthotoxin, suggesting that insertion of GluA2-lacking AMPA receptors is required for leptin-induced LTP. AMPA receptor trafficking events also underlie the effects of leptin at adult TA-CA1 synapses, as removal of GluA2-lacking AMPA receptors is key for induction of LTD by leptin [30]. Together, these findings indicate that the ability of leptin to regulate the movement of AMPA receptors to and away from hippocampal synapses is key for maintaining the bi-directional effects of leptin on excitatory synaptic efficacy at TA- and SC-CA1 synapses (Figure 2). 

The cellular mechanisms responsible for the insertion of GluA2-lacking AMPA receptors in response to leptin has been studied in detail. Thus, using a combination of electrophysiology and immunocytochemical approaches, Moult and colleagues (2010) observed that the leptin-driven increase in GluA1 surface expression was accompanied by an elevation of the intracellular levels of the second messenger, phosphoinositide 3,4,5-trisphosphate (PIP_3_). It is well known that PIP_3_ levels are regulated by the enzyme, PI 3-kinase which promotes conversion of phosphoinositide-4,5-bisphosphate (PIP_2_) into PIP_3_. The phosphatase and tensin homolog (PTEN) also regulates intracellular PIP_3_ levels, as PTEN activity counteracts the actions of PI 3-kinase, by dephosphorylating PIP_3_, which in turn promotes the generation of PIP_2_. Inhibition of the phosphatase, PTEN, not only mirrored the effects of leptin but it also occluded the ability of leptin to elevate PIP_3_ levels, and to traffic GluA1 to hippocampal synapses, suggesting that leptin-driven inhibition of PTEN underlies both events. 

## 4. Leptin and the Ageing Brain

Numerous clinical studies have shown that during the human ageing process, there is a gradual decline in the functionality of metabolic systems. This has implications not only for the metabolic function of peripheral tissues, but as metabolic hormones have widespread actions in the CNS, deteriorations in metabolic function may impact not only overall brain health but also influence the risk of age-related neurodegeneration later in life. In this respect, there is good evidence supporting the notion that Alzheimer’s disease (AD) is related to dysfunctions in brain glucose metabolism which can lead to progressive and brain-region specific neuronal degeneration [35,36]. A growing body of evidence indicates that alterations in leptin function are also correlated with AD. Indeed, clinical studies have identified lifestyle factors, like diet and body weight, as having a significant impact on development of AD. In this respect, there is considerable evidence that weight gain or an obese phenotype in middle age increases AD risk [37,38,39,40]. In contrast, weight loss in elderly individuals is associated with a higher likelihood of AD [41,42,43], suggesting that body weight has a complex yet paradoxical effect on AD risk. It is interesting to note that the risk of developing other neurodegenerative conditions, like amyotrophic lateral sclerosis (ALS), is also influenced by body weight status [44,45]. 

The link between body weight and neurodegeneration risk suggest a central role for adipose tissue, and adipokines like leptin, in determining clinical outcome across a spectrum of CNS disorders including AD. In support of leptin’s role in AD, the leptin system is altered in AD such that abnormally high as well as low leptin levels are reported in AD sufferers [46,47]. Moreover, in prospective studies, a lower incidence of AD was detected in individuals who had high circulating leptin levels in the absence of an obese phenotype [48]. Comparable correlations between leptin and neurodegeneration risk have been demonstrated in rodent models of AD, with APPSwe and CRND8 mouse models both displaying significantly lower circulating leptin levels than age-matched wild-type animals [49,50]. However, a complex picture is emerging of leptin’s role in AD as recent clinical studies have failed to detect significant alterations in circulating leptin levels in AD patients or a direct correlation between leptin and cognitive decline [39,51,52]. A range of confounding factors, such as gender, diet, and exercise may have contributed to the reported discrepancies in these studies. As metabolic function involves close interplay between leptin and other endocrine hormones, like ghrelin and insulin, it is likely that the relative levels of these hormones, as well underlying metabolic disorders, like diabetes, all influence the overall risk of AD. Consequently, monitoring the plasma levels of leptin in isolation, may not be the best determinant of AD risk. 

In addition to age-related changes in metabolic function, alterations in neuronal responsiveness to metabolic hormones with age have been observed. Indeed, numerous studies have identified significant reductions in the insulin system, including downregulation of receptors and associated signalling cascades, as a result of the ageing process [53,54]. In accordance with this, deterioration in leptin responsiveness and leptin receptor functionality are also associated with the ageing process. For instance, studies in hypothalamic neurons demonstrated that the ability of leptin to regulate satiety is markedly reduced in aged compared to adult rats [55] and altered hypothalamic leptin-responsiveness is linked to diminished capacity to stimulate STAT3 signalling [56]. Significant changes in leptin function are also observed in the hippocampus with age. Thus, studies comparing the age-related effects of leptin on hippocampal synaptic function detected marked variance in hippocampal responsiveness to leptin between 3–4 month and 12–14 month of age, such that the magnitude of LTP evoked by leptin at 12–14 month was ~50% less than leptin-induced LTP at 3–4 month of age [24]. As NMDA receptor activation is a pre-requisite for leptin-induced LTP, and it is known that NMDA receptor function declines with age, it is feasible that a decrease in the ability of leptin to enhance NMDA receptor function contributes to the attenuated leptin-responsiveness with age, although this remains to be determined experimentally. 

### 4.1. Neuroprotective Actions of Leptin

Comparative studies performed in leptin-sensitive and leptin-deficient rodents were some of the first to uncover possible neuroprotective actions of leptin. Significant CNS changes including a fall in brain weight were detected in *ob*/*ob* mice relative to wildtype littermates, suggesting decreased neuronal viability is associated with a lack of leptin [14]. This role of leptin was further verified by the complete reversal of these brain abnormalities after treatment with leptin. Numerous studies have since confirmed a neuroprotective role for leptin, with leptin-based treatments linked to enhanced survival of neurons as well as reduced neuronal apoptosis in response to various toxic agents [57,58,59]. Recent evidence indicates that leptin-driven improvements in mitochondrial function may contribute to leptin-driven increases in neuronal viability [60]. Accumulating evidence indicates that leptin displays neurotrophic properties, but this may be restricted to specific neuronal populations and brain regions. For example, leptin is reported to selectively promote neurite outgrowth from cerebellar purkinje, but not granule, cells [61]. 

The neuroprotective effects of leptin extend to various models of CNS-driven disease including models of ischaemia [62,63,64]. Indeed, after transient periods of ischaemia, treatment with leptin is reported to reduce the size of cerebral infarcts and degree of brain oedema [65]. Leptin treatment also promotes an increase in the levels of ATP and p-Akt, at the same time as reducing the levels of lactate dehydrogenase, which will ultimately lead to overall enhanced neuronal cell survival in ischaemic stroke models. 

**Figure 3:**
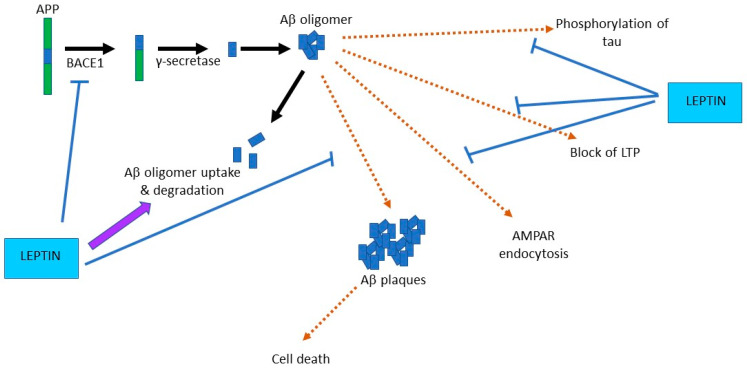
Leptin prevents the aberrant toxic effects of β-amyloid (Aβ) at synapses. Cleavage of amyloid precursor protein (APP) by the sequential actions of Beta secretase-1 (BACE1) and γ-secretase generates Aβ protein that readily aggregates into oligomeric forms of Aβ. Acute exposure to Aβ oligomers promotes toxic actions at synapses that include tau phosphorylation, AMPA receptor endocytosis, and block of hippocampal LTP. Chronic exposure to Aβ leads to plaque formation and enhanced cell death. Leptin prevents the acute effects of Aβ on synaptic function, and it limits Aβ production and overall brain load, by reducing BACE1 activity, and promoting degradation of Aβ.

Protective actions of leptin have been observed in several neurodegenerative models that replicate key pathological features of Parkinson’s disease [57,66,67] and AD [50,68,69]. In AD, build-up of toxic β-amyloid (Aβ) is known to promote formation of amyloid plaques, and that accumulation of Aβ involves proteolytic processing of amyloid precursor protein (APP), with specific generation of the toxic forms of Aβ driven by sequential β- and γ-secretase cleavage of APP. Expression of β- and γ-secretase is downregulated by leptin, which would lead to reduced levels of toxic Aβ, due to enhanced APP cleavage by α-secretase [70,71,72]. Studies in neuronal cells have demonstrated that leptin directly interferes with Aβ levels via blocking β-secretase action [72]. Leptin also diminishes the extracellular levels of Aβ by driving Aβ uptake into cells [73] (Figure 3). Furthermore, treatment of an AD rodent model with leptin gives rise to a significant reduction in brain amyloid load [68]. Introduction of leptin into APP/PS1 mice using lentiviral gene therapy approaches results in an overall decrease in Aβ accumulation as well as recovery of some of the AD-related synaptic deficits [74,75]. Together, these studies indicate that leptin not only interferes with APP-dependent generation of Aβ but it also limits its toxic accumulation in neurons.

Another key pathological feature of AD is the formation of neurofibrillary tangles, composed of hyper-phosphorylated tau. One of the key enzymes that regulate the phosphorylation status of tau is glycogen synthase kinase 3β (GSK3β), a serine/threonine protein kinase, which is inhibited by PI 3-kinase. Recent studies indicate that leptin inhibits activation of GSK3β, via stimulation of PI 3-kinase, and that activation of this LepR-signalling pathway limits tau phosphorylation [73,76]. Exposure to leptin is also reported to decrease the levels of phosphorylated tau (p-tau) in rodent models of AD [73]. The ability of leptin to regulate the levels of p-tau is supported by histological studies as raised levels of p-tau are observed in cortical tissue from Zucker *fa*/*fa* rats compared to Zucker lean rats, suggesting that leptin-insensitivity boosts tau phosphorylation [77]. Transgenic mice that display resistance to leptin also exhibit elevated levels of p-tau compared to wild-type controls [78]. These findings provide further verification that lack or insensitivity to leptin enhances phosphorylation of tau. 

### 4.2. Leptin Prevents Aβ-Driven Impairments at Hippocampal Synapses

It is well established that acute exposure to oligomeric Aβ_1-42_ has damaging effects at excitatory synapses. Indeed, one consequence of treating brain slices with Aβ_1-42_ is that there is inhibition of LTP at hippocampal SC-CA1 synapses [79]. Additionally, activity-dependent induction of LTD is facilitated in slices exposed to Aβ_1-42_ and this process is thought to involve the Aβ_1-42_-driven removal of synaptic AMPA receptors [80]. Recent electrophysiological studies indicate that prior treatment of acute hippocampal slices with leptin prevents these aberrant effects of Aβ such that the ability of Aβ_1-42_ to inhibit LTP and to facilitate LTD is blocked by leptin [77]. Moreover, exposure of cultured hippocampal neurons to leptin prevents Aβ_1-42_-driven removal of GluA1-containing AMPA receptors from hippocampal synapses [77]. PI 3-kinase is implicated in the protective actions of leptin at synapses as the beneficial effect of leptin in preventing Aβ_1-42_ inhibition of LTP is blocked by pharmacological inhibition of PI 3-kinase. The capacity of leptin to limit Aβ_1-42_-driven AMPA receptor endocytosis is also hindered by PI 3-kinase inhibitors [77]. As exposure to Aβ_1-42_ promotes cleavage of Akt, which in turn limits inhibition of GSK-3β [81], it is feasible that leptin counteracts this chain of events via activation of PI 3-kinase and subsequent inhibition of GSK-3β. In support of this possibility, administration of pharmacological inhibitors of GSK-3β not only replicate but also occlude the effects of leptin [73].

### 4.3. Leptin Improves Hippocampus-Dependent Learning and Memory

It is widely accepted that activity-dependent synaptic plasticity is likely to be a key cellular process involved in hippocampal-dependent learning and memory [82]. As significant evidence now suggests that leptin facilitates the same cellular events that underpin hippocampal learning and memory, it is likely that leptin also influences hippocampus-dependent behaviours. Indeed, several studies have observed improved performance in various hippocampus-dependent learning and memory tasks in rodents treated with leptin. Thus, the ability of rodents to perform spatial memory tasks in the Morris water maze improves after intravenous administration of leptin [83], whereas insensitivity to leptin is associated with impaired performance in hippocampus-specific memory tasks [19,20]. Dietary changes that lead to leptin resistance in rodents are also associated with significant impairments in novel object recognition tasks [84]. Conversely, peripheral administration of leptin enhances the ability of mice to recognize novel objects [85].

Numerous studies support the notion that the detrimental effects of Aβ_1-42_ on hippocampal synaptic function result in cognitive impairments, and these events closely correlate with the memory deficits and impaired cognitive function that occur in human cases of AD. In accordance with leptin’s ability to prevent the aberrant actions of Aβ_1-42_ at hippocampal synapses, treatment with leptin also improves cognitive function in rodent models of AD. Thus, direct administration of leptin into the hippocampal formation boosts memory retention in SAMP8 mice, as evidenced by improved performance in T-maze and passive avoidance behavioural paradigms [49]. Performance in novel object recognition assays is also improved in CRND8 transgenic mice in response to leptin treatment [73]. Furthermore, deficits in spatial memory due to intracerebroventricular application of toxic Aβ_1-42_ are attenuated in rats following treatment with leptin [69]. In addition, lentiviral administration of leptin directly into brain ventricles counteracts the reported impairments in hippocampal-dependent memory in APP/PS1 transgenic mice [75]. Consequently, there is now good evidence from studies in wild-type animals and in various disease models that leptin augments cognitive function and specifically enhances hippocampus-driven memory processes.

## 5. Targeting the Leptin System as a Novel Therapeutic in AD? 

Leptin is known to be safe for human use and it is already licensed as an anti-obesity treatment. Clinical studies where leptin has been used to treat obesity have identified that leptin also exhibits beneficial cognitive properties, which parallel the cognitive-enhancing actions of leptin in rodents. Thus, in functional brain imaging studies of individuals with congenital leptin deficiencies, Matochik and colleagues [86] found that leptin treatment enhanced gray matter volume, which is indicative of improved cognitive function. Moreover, cognitive deficits in a 5-year-old child, who was unable to produce leptin due to a leptin (*ob*) gene mutation, also markedly improved in response to a leptin treatment strategy [87]. These studies reiterate that leptin-based therapies are safe for use in humans, but also that leptin can readily penetrate the CNS and target key brain regions, like the hippocampus, that are key for higher cognitive functions. Although there is now good experimental evidence supporting the beneficial effects of leptin in AD models, studies in human AD patients have yet to be performed and thus the potential therapeutic efficacy of leptin in AD remains to be examined in a clinical setting. However, given what is already known about the central actions of leptin, it is feasible that not all AD patients would respond to leptin-based therapies. Consequently, identifying patients who would likely benefit would be crucial in clinical trials. Thus, individuals with midlife obesity and leptin resistance may not respond, whereas treatment with leptin may offer significant benefit to patients with low circulating leptin levels. 

One area that offers new hope for an AD therapy is in the possible use of leptin-based peptide molecules that mimic the actions of leptin. In this respect, fragments of the whole leptin molecule have already been identified as having anti-obesity actions in various studies [88,89]. Recent studies have extended these findings to show that one specific leptin fragment, namely, leptin_116-130_, not only displays hippocampal bioactivity but it replicates the cognitive-enhancing actions of leptin by facilitating the induction of hippocampal LTP, and by driving insertion of GluA1-containing AMPA receptors into synapses [85]. In addition, mice perform much better in episodic-like memory tasks after peripheral administration of leptin_116-130_, compared to mice treated with vehicle. Moreover, leptin’s neuroprotective actions are mirrored by leptin_116-130_, as treatment with this leptin fragment prevents the detrimental effects of Aβ_1-42_ as the ability of Aβ_1-42_ to prevent hippocampal LTP induction and to promote internalization of GluA1 subunits are blocked by leptin_116-130_ [85]. Although these findings are promising, further pre-clinical studies in rodent models of AD are required, before verifying that leptin_116-130_, or related peptides are suitable clinical targets. 

## 6. Protective Actions of Leptin in Other Neurodegenerative Diseases

In accordance with the proposed link between metabolic imbalance, mid-life obesity and risk of AD, several other neurodegenerative conditions are also associated with altered metabolic status, including PD, Huntington’s disease (HD) and multiple sclerosis (MS) [90,91,92,93]. Furthermore, the plasma levels of leptin are significantly reduced in HD and PD patients [94,95,96], suggesting that functioning of the leptin system is modified in these CNS disorders. Parallel changes in leptin function are also observed in rodent models of PD, with attenuated leptin levels and metabolic dysfunction key reported features of mutant α-synuclein mice [97]. In relapsing MS patients, elevated levels of LepR expression as well as LepR signalling via STAT3 have been detected in T cells and monocytes, suggesting a possible role for leptin in MS relapse [98]. 

Recent studies also suggest a link between leptin and degenerative conditions affecting motor neurons, as leptin levels are elevated in ALS patients [99] and in children with spinal muscular atrophy [100]. In addition, population-based studies of German ALS patients have revealed a relationship between leptin levels and the risk of developing ALS [101]. Moreover, recent studies indicate that reducing leptin levels improves lifespan and reduces motor neuron degeneration in mutant superoxide dismutase 1 (SOD1) mice, suggesting that altering leptin levels mitigates motor neuron disease progression [102]. 

## 7. Conclusions

The neuronal actions of the metabolic hormone leptin are known to extend beyond the hypothalamus and energy homeostasis, to the hippocampal formation where leptin boosts cognitive function. In addition to regulating classical SC-CA1 synapses that play a role in spatial memory, TA-CA1 synapses have been identified as an important target for leptin. As the TA input to CA1 neurons is implicated in episodic memory, the ability of leptin to regulate this synaptic connection is likely to also influence episodic memory processes. It is widely recognized that body weight, and dysfunctions in metabolic systems, have a role to play in development of AD, and evidence from clinical studies has uncovered correlations between the plasma levels of leptin and the risk of AD. In addition, accumulating evidence indicates beneficial cognitive and neuroprotective effects of leptin and leptin fragments in a variety of different rodent models of AD. Consequently, targeting the leptin system may offer a novel therapeutic avenue for the development of an effective AD treatment. However, in the absence of clinical assessment, it is unclear how effective leptin would be. It is likely that a leptin-based therapy would not benefit individuals displaying leptin resistance, nevertheless, there are potential therapeutic benefits of leptin in AD patients with low leptin levels. Furthermore, these benefits are not restricted to AD as emerging studies suggest that other neurodegenerative diseases, like ALS, are also associated with metabolic imbalance and abnormalities in the leptin system. Further studies are needed to examine thoroughly the therapeutic potential of the leptin system in AD and other neurodegenerative disorders.
